# An Exploratory Study to Understand Nonprofit Organizations’ Crisis Leadership Competencies: A Portuguese Analysis on COVID-19

**DOI:** 10.1007/s10672-023-09438-5

**Published:** 2023-02-08

**Authors:** Salete Esteves, Lara Santos, Luisa Lopes

**Affiliations:** 1 Instituto Politécnico de Bragança, Campus de Santa Apolónia, 5300-253 Bragança, Portugal; 2 Centre for Tourism Research, Development and Innovation - CiTUR, Guarda, Portugal; 3 Universidade Lusófona, TRIE, Porto, Portugal

**Keywords:** Nonprofit organizations, Crisis management, Organizational leadership competencies, Exploratory factor analysis, COVID-19

## Abstract

The purpose of this study is to investigate the factors that affect the Nonprofit Organization’s (NPO) competencies and leadership in a crisis situation, specifically in the Portuguese NPOs during COVID-19. Adopting an interdisciplinary perspective, this article integrates crisis management literature with leadership literature. Highlighting the leadership perspective of both intermediate employees (technical director) and top management (executive director), this research aims to advance knowledge on the main organizational leadership competencies that NPOs need to have to better cope with crises. One hundred and seventy-four (174) NPOs representatives voluntarily participated in the study which involved a survey questionnaire based on a five-point Likert scale for 23 items included in the questionnaire. Principal component analysis using varimax rotation was applied to reduce the number of variables. Reliability tests were performed to assess the items included in the questionnaire. Tests included test-retest reliability, Cronbach alpha, and split-half reliability coefficients. Results show that the most important factors that improve the capability of a NPO to cope with a crisis are: respond to all stakeholders through accountability, plan based on identification of vulnerabilities, and build a foundation of trust through communication. Additionally, it is possible to suggest that staff and top management perceive crisis and leadership competencies differently. It is possible to conclude, that these factors can be used as important lines of action to structure the sustainable development and planning of NPOs’ strategies of other similar crisis to come in the future. Findings, the implications of this work, and avenues for future NPOs crisis management and leadership research are addressed.

## Introduction

COVID-19 brought change at an unprecedented scale and pace that challenged the Nonprofit Organizations (NPOs) world in many ways. To respond to emerging and rapidly changing needs, these organizations were forced to adapt leadership strategies and governance structures (McMullin & Raggo, 2020). Management capabilities have been extensively explored in public and private organizations, contrary to those of NPOs (Adro & Leitão, 2020; Bish & Becker, 2016). Concepts such as leadership and the skills needed to successfully face crises are substantially discussed in the literature (Gilstrap et al., 2016; McMullin & Raggo, 2020). However, less academic attention has been specifically paid to how NPOs leadership contributes to organizational responses to crises (Gilstrap et al., 2016; McMullin & Raggo, 2020). Leadership in NPOs is considered complex in terms of behaviors, linkages, group needs, and mission objectives (Gilstrap et al., 2016). When the diversity and responsibility burdens of NPOs leadership are mixed with the additional pressures of organizational crises, the complexity expands considerably (Gajewski et al., 2011). In part, this is a consequence of the literature on NPOs governance being almost exclusively concerned with top management structures (Cornforth, 2012; McMullin & Raggo, 2020). Furthermore, current research ignores how NPOs leaders understand their organizations during crises (Gilstrap et al., 2016).

Therefore, we must question if the existing knowledge, theories and models of organizational change and the leadership and management competencies performed by top management can be sufficient to explain the pace and scale of change required by the COVID-19 pandemic, or other types of crises. In this respect, this article intends to support NPOs crisis management efforts by developing knowledge at the level of the needed management and leadership competencies, when applied to the third sector. Evidence of competency models in the non-profit context is scarce and a competency-based approach in the NPOs context will help to develop a model that points to the underlying characteristics of an individual leading to superior performance in a crisis situation (Meduri, 2021).

To fill in the aforementioned research gap, this study aims to deepen knowledge about crisis competencies and leadership in NPOs, by answering the following question: What are the key factors on organizational and leadership competencies in times of crisis that NPOs should develop? Based on the six essential competencies for crisis leadership presented by James and Wooten (2005), an online questionnaire was developed, and results will be discussed.

The article proceeds as follows. First, with the research background in mind, it deepens knowledge on leadership and crisis management. By doing so, knowledge of this field can be adapted to the specific context of NPOs. Second, it presents the methods carried out in this phase of the study and clarifies the data collection process. It also examines and discusses data and results obtained in this field study. Finally, conclusions and implications for further research are drawn.

## Crisis Leadership Competencies

A crisis, in the crisis management literature, has been identified according to typologies, situations, context, phases and decision making (Coombs & Laufer, 2018; Hileman, 2022; James & Wooten, 2005; Mitroff et al., 2006). Charles Hermann, in 1963, was one of the first authors to write about crises and his concern was to analyze the consequences that certain disruptive phenomena had on the viability of organizations. This author defined a crisis as something that threatens the fundamental values of the organization, allows only a limited period of time for decision making, is unexpected by the organization and originates in the relevant environment of the organization (Hermann, 1963).

Crisis management is currently conceptualized as a process model that views the crisis and organizational response as a phenomenon that follows a certain chronological order (Fener & Cevik, 2015; James et al., 2013, p. 697) define crisis management “as the process where the indicators of crisis are obtained and assessed for the risk of a potential crisis and where necessary measures are taken and applied in order to experience minimum loss in a state of crisis”. Researchers tend to investigate the stage of crisis preparedness and planning, crisis types, organizational culture, crisis teams, management groups, organizational learning and post-crisis development, and future crisis preparedness with implementation of corrective actions (Coombs & Laufer, 2018; DuBrin, 2013; Mitroff et al., 2006; Pearson & Clair, 1998). Additional studies are dedicated to understanding how managers learn to deal with crises based on their mistakes, lessons learned, and best practices from other organizations and communities (Deverell, 2013; Stern, 2013).

The concept of crisis leadership emerged from crisis management research (Bhaduri, 2019) and can be defined as a process, and the ability to demonstrate a core set of behaviors in a complex and dynamic environment (James & Wooten, 2005). Taking the work developed by DuBrin (2013) as an example, it is possible to conclude that management behaviors during a crisis include: making clear, direct, and unambiguous decisions, acting with resilience and demonstrating compassion and flexibility. The increasing development of these studies allow us to perceive the crisis from the perspective of management, focusing on the behavior of management and not leadership or competencies (James & Wooten, 2005), and tend to emphasize experiences in public organizations and for-profit organizations (Gilstrap et al., 2016; Meisler et al., 2013).

It is the leader’s responsibility to respond to threats and uncertainties arising from crises (Demiroz & Kapucu, 2012). Still, perhaps the most prominent role of a leader during a crisis is to assert responsibility for communication (Urick et al., 2021). According to Fearn-Banks (2017) and Frandsen and Johansen (2009) crisis communication is the dialogue or communication processes between the organization and the public before, during and after an event or situation that the organization and its stakeholders interpret as a crisis. Following the same line of thought, Sheehan and Quinn-Allan (2015) mention that crisis communication is a process that organizations employ for good crisis management. Crisis communication is a “set of practices associated with public relations and used by management to address public concerns, coordinate resources, reduce harm, and improve social understanding of risk so that stakeholders are able to respond collaboratively, and responsible in crisis situations” (Gilstrap et al., 2016, p. 5).

In this respect, the skills of leaders and the way they relate to all the organization’s stakeholders influence how well the organization can withstand the challenges it faces. In fact, as James and Wooten (2005, p. 141) have observed, “what differentiates companies that thrive after a crisis from those that don’t is the leadership displayed throughout the process”. However, some researchers (e.g., Seeger, 2006; Ulmer, 2012) point out that there is a need to recognize the role of critical thinking leadership in a crisis.

Most crisis leadership studies, whether in management, communication, or public administration, focus on crisis responses (Fener & Cevik, 2015). Other studies develop the key elements of crisis leadership in a variety of ways. Boin et al. (2005) point out five essential tasks for leadership – making sense of the crisis, making decisions to deal with the crisis, framing and giving meaning to the crisis for stakeholders, ending the crisis to restore normality, and guiding the organization to learn from the crisis. On the other hand, James and Wooten (2005) linked crisis management to leadership by identifying core competencies of leaders in different phases of crisis. According to their study, there are six relevant competencies: building a foundation of trust, creating a new corporate mindset, identifying the (not so) obvious firm vulnerabilities, making wise and rapid decisions, taking courageous action, and learning from crisis to effect change (James & Wooten, 2005). Competencies, unlike skills, include behavioral attributes of an individual, which are observable, measurable and trainable and that allow him to achieve a higher level of performance required at work (Meduri, 2021). Competency is the process of executing a task at a high level and may lead to new standards of performance (Perry, 2019). Over the years, many organizations and researchers have proven a significant and positive change in the performance of organizations that have adopted a competency model (Martone, 2003; Stephen & Neville, 2012). A part of the investigation was carried out to identify the competencies required by logistics and supply chain professionals in business organizations (Gowen & Tallon, 2003; Knight et al., 2005; Mangan & Christopher, 2005; Sohal, 2013; Thai et al., 2011).

There is not enough empirical data to look specifically at the role of leadership and competencies in crisis management (Jin et al., 2017). Perhaps, unsurprisingly, much of the existing literature is grounded in databases and case studies of real crises, expert opinions, and leaders’ self-reflections (e.g. Boin et al., 2010; Demiroz & Kapucu, 2012; James & Wooten, 2005; Mutch, 2020; Urick et al., 2021). In these studies, researchers generally analyze factors that drive crisis situations, the actions and leadership styles demonstrated to face challenges, and whether these leadership responses can be considered effective or ineffective (Caringal-Go et al., 2021).

## NPOs Crisis Leadership Competencies

NPOs play an increasingly influential role as a catalyst for new approaches as well as crucial actors in social and economic life (Salamon & Anheier, 1992). These organizations are a vital pillar of the welfare state and territorial cohesion, representing a considerable percentage of the gross national product and a relevant part of the economy of many countries (Adro & Leitão, 2020). Despite their status, and the growing interest of society and academics, NPOs are one of society’s least understood organizations (Waters, 2014). Moreover, NPOs are not immune to crises (Jordan et al., 2016; Schwarz & Pforr, 2011; Sisco, 2012; Spillan, 2003; Wrigley et al., 2003), and must prepare for unforeseen events that could put the sustainable implementation of their activities at risk (Santos & Lopes, 2021; Willems, 2016; Drucker, 1995) has suggested that even though nonprofits struggle to secure adequate resources, it is oftentimes a lack of competency that drives organizational failure. Organizational competencies across nonprofit executives, board members, and management could lead to organizational effectiveness (Perry, 2019). With societal pressures demanding that nonprofit executive leaders start emphasizing performance outcomes, efficiency, and evaluation much more so than in the past, an examination of organizational competencies in a nonprofit setting is desperately needed (Drucker, 1995; Perry, 2019). However, evidence of competency models in the non-profit context is minimal (Meduri, 2021). Particularly disconcerting is the lack of assessment of the NPO leader in the context of the crisis, given that nonprofit organization leaders are a heterogeneous group inserted in various spaces and functions within their organizations (McClusky, 2002) that build and maintain necessary organizational relationships - for the success of NPOs – especially during times of crisis (Gilstrap et al., 2016). Whether in the form of community leaders and/or individuals who build ties between other NPOs and the government, the most well-known leadership roles in NPOs (members of their governing bodies, chair, board of directors, etc.) exert influence over executive leaders and influential stakeholders (Burnett, 1998; Gilstrap et al., 2016; Jeong & Kearns, 2015; King, 2002). In addition, NPOs leaders are focused on building and maintaining relationships among an array of stakeholders (i.e., volunteers, audiences, partners, customers) to fulfill the needs of their organizational mission (Chikoto et al., 2013; Gilstrap et al., 2016; Jeong & Kearns, 2015). NPOs board members can play a dual role: operating as managers when they focus on establishing decision-making structures and monitoring the work of executive members, and as leaders in developing a strategic vision for the organization and guiding the executive director’s work (McMullin & Raggo, 2020).

Explicit attention devoted to the role of NPOs leadership in times of crisis is scarce (Gilstrap et al., 2016; McMullin & Raggo, 2020), although there is extensive literature related to the roles, duties, and characteristics of NPOs top management (Brown & Chao Guo, 2010; Cagney, 2018; McMullin & Raggo, 2020). Responding to this research gap, the present study analyzes the core competencies and behaviors of NPOs leaders in crisis situations.

## Methods

**Fig. 1:**
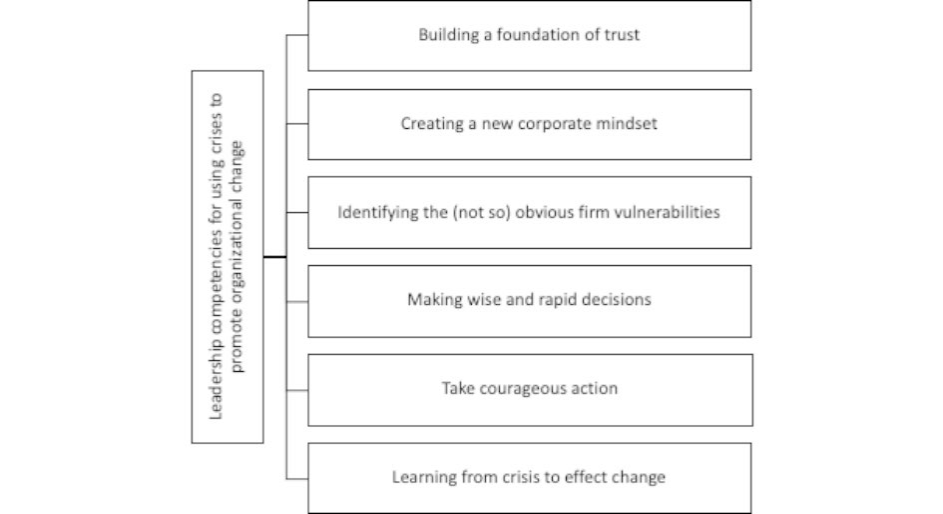
Leadership competencies for using crises to promote organizational change. (Source: Based on James and Wooten’ (2005) seminal work)

This study assumes a quantitative approach and its purpose is to build knowledge identifying the core organizational competencies of NPOs in times of crisis. Inspired by James and Wooten (2005), this study explores the concept of NPOs crisis competencies and leadership conceptualized as a multi-faceted construct, which includes a large number of attributes such as vulnerability identification, trust, communication, accountability to all stakeholders, strategic guidance, decision-making, courageous action, and learning (Cohen et al., 2017; Fener & Cevik, 2015; James & Wooten, 2005; Urick et al., 2021). This study intends to reduce and refine the core competencies, analyzing and confirming, or not, the James and Wooten (2005) proposal, adapting it to NPOs setting (see Fig. 1).

Because the world is still in a pandemic situation, this study focuses on the pre-crisis and crisis phases, and is based on the literature review. Table 1 summarizes the main methodological elements used in the collection of quantitative data.

**Table 1:** Synthesis of online survey

Temporal basis	Cross-Section
**Unit of analysis**	NPOs executive and technical director
**Sampling**	Convenience
**Sample**	168
**Data collection**	Questionnaire survey available online
**Date**	June to September 2021
**Data analysis**	Univariate and multivariate

Source: Research data

### Sample

The population of this study is composed of 1124 Portuguese NPOs, of a database (Gabinete de Estratégia e Planeamento, 2020) of social economy organizations of mainland Portugal from which the data was collected, resulting in a convenience sample. The survey made of Portuguese NPOs received a total of 174 valid responses out of 1124 total NPOs (15.5%), but six responses were not considered as the respondents were not technical directors or executive directors (n = 168). Similar studies have similar valid responses (e.g. Adro et al., 2022, n = 135). These were essentially asked about their perception of the institution’s level of preparedness and reaction to COVID-19 pandemic, in a major study that included leadership competencies. Additionally, for each institution and respondent, social and demographic profiles were assessed. Data collection started in June and ended in September 2021.

### Data Collection Tool

The collection of data was performed via an original questionnaire developed and distributed on the internet using e-mails of institutions and social media (five NPOs Facebook groups). It was made available in an electronic medium (Lime Survey platform). The data collection tool was developed according to the literature review and considering what could be applied to NPOs setting. Within the scope of tailoring the survey to the specific characteristics of the third sector, as well as ensuring harmonization, we used the words “institution” and “beneficiary”. A pre-test of the questionnaire was carried out with six specialists in the field with more than six years of experience, who suggested small changes. The total instrument (of the major study) consisted of four parts: socio-demographic characterization, pre-crisis, crisis, and post-crisis. Socio-demographic data included a brief characterization of the respondent (gender, age, education, function, and experience) and the institution (number of employees, and volunteers, before and during COVID-19, intervention area, social answers, district, and legal form).

The organizational leadership competencies were delineated based on one seminal work (James & Wooten, 2005). Items generated accounted for 23 (statements), measured using a five points Likert scale ranging from 1 = strongly disagree to 5 = strongly agree. The point of equilibrium on the scale was 3 = neither agree nor disagree.

## Data Analysis and Results

Data entry, processing and results analysis were performed using the Statistical Package of the Social Science (SPSS) Software, version 26.0. The final sample consisted of 168 individuals. Results were analyzed using descriptive tables and cross-tabulation and using the Chi-square test of independence, Mann-Whitney U test and Factor analysis, where the significant effect was equal or less than 0.05. The 5% level of significance was used throughout the statistical analysis for all relevant tests.

The demographic characteristics of the resulting sample (Tables 2 and 3) indicate female as the dominant gender (76.2%).

**Table 2:** Characteristics of the sample of respondents

	Technical Direction		Executive Direction		
Variables	Frequency	%	Frequency	%	χ2
**Gender**					12,3*
Male	20	16,4	19	42,2	
Female	102	83,6	26	47,8	
**Education**					11,85*
Primary and secondary	0	0	4	8,9	
Higher education (Bachelor degree)	69	56,1	26	57,8	
Post- graduation/MBA/Master´s/PhD	54	43,9	15	33,3	

Source: Research data *p-value < 0.01

Respondents were, on average, 43 years old, 56.6% had higher education (bachelor’s degree) and 41.4% Postgraduate/MBA/Master’s/PhD. Regarding the position held in NPOs, 26.8% of respondents are in top management as executive directors (president of charitable organizations, general manager, administrator…) and 73.2% as technical directors^1^. The average number of years of respondents in these NPOs is 13 and the average number of years of experience in the current position is 11.

Comparing the technical direction and the executive direction, statistically significant differences were found between the two groups in terms of gender and education. The chi-square test of independence was used, and it was observed that there are more women in the technical direction group (83.6% versus 47.8%) and more individuals with Postgraduate/MBA/Master’s/PhD in the same group (43.9% versus 33.3%), compared to the executive direction, where the sample includes less women and individuals with lower education (bachelor’s degree).

Table 3 presents some characteristics of the NPOs participating in this study. Regarding the district where the institution is located, responses were obtained from all districts of mainland Portugal. As for the area of intervention, most work in an institution whose area of intervention involves the elderly (62.6%) and children and youth (51.6%). The average number of employees before the pandemic was approximately 58.95 ± 70.26 and 61.79 ± 77.02 during the pandemic. The average number of volunteers before the pandemic was 6.03 ± 16.99 and changed to 4.79 ± 17.44 during the pandemic.

**Table 3:** Characteristics of the NPOs

District	Frequency	%
Aveiro	9	5,4
Beja	4	2,4
Braga	15	8,9
Bragança	13	7,7
Castelo Branco	3	1,8
Coimbra	3	1,8
Évora	4	2,4
Faro	6	3,6
Guarda	6	3,6
Leiria	10	6,0
Lisboa	18	10,7
Portalegre	4	2,4
Porto	17	10,1
Santarém	9	5,4
Setúbal	12	7,1
Viana do Castelo	13	7,7
Vila Real	9	5,4
Viseu	13	7,7
**Intervention area**		
Children and Youth	80	51,6
Children and Youth with Disabilities	24	15,5
Children and Youth in Critical Situation	28	18,1
The Elderly	97	62,6
Adults with Disabilities	34	21,9
People in Dependency Situation	20	12,9
The Homeless	5	3,2
Family and Community in General	37	23,9
People with HIV/AIDS and Their Families	6	3,9
Drug Dependent Person	8	5,2
Victims of Domestic Abuses	7	4,5
**Legal form**		
Association	79	47,0
Parish and Social Center	31	18,5
Foundation	13	7,7
Religious Institution	12	7,1
Holy House of Mercy	33	19,6
Average number of total employees (before COVID-19) (Mean ± SD*)	58,95 ± 70,26	
Average number of total employees (during COVID-19) (Mean ± SD*)	61,79 ± 77,02	
Monthly average number of volunteers (before COVID-19)(Mean ± SD*)	6,03 ± 16,99	
Monthly average number of volunteers (during COVID-19)(Mean ± SD*)	4,79 ± 17,44	

Source: Research data *SD - standard deviation

The following 23 items about organizational leadership competencies were subjected to the exploratory principal component analysis. This is a statistical procedure used to reduce a large number of items to a small number of factors/components. As a first step, we study the reliability (internal consistency) of the items. Table 4 shows the reliability analysis of the 23 items. The mean and standard deviation of the items ranged between the highest for clients or beneficiaries trust in the institution’s services 4.44 ± 0.65 and the lowest for during a crisis, the institution tends to deny the accusations or to say the least is 2.18 ± 1.20. The results of this analysis showed the homogeneity of items.

**Table 4:** Total reliability analysis of items of NPOs crisis leadership competencies

Item	Mean	SD	Corrected item – Total correlation	Alpha if item deleted
CL1-The leadership team always communicates openly and honestly.	4,34	0,79	0,70	0,90
CL2-The internal and external communication is explicit.	4,18	0,80	0,73	0,90
CL3-Internal and external communication is sufficient.	3,92	0,93	0,69	0,90
CL4-There is sharing of relevant information	4,26	0,76	0,70	0,90
CL5-Employees feel safe in the work environment	4,18	0,78	0,64	0,90
CL6-Clients or beneficiaries trust in the institution’s services	4,44	0,65	0,62	0,90
CL7-The institution’s partners expect cooperative actions and intention	4,10	0,84	0,52	0,91
CL8-The institution meets the needs of all stakeholders	3,96	0,81	0,68	0,90
CL9-The institution responds to all stakeholders	4,03	0,82	0,63	0,90
CL10-The institution is transparent and accountable to all stakeholders	4,27	0,79	0,70	0,90
CL11-The institution considers all perspectives in decision making	4,18	0,76	0,75	0,90
CL12-The institution has identified all its vulnerabilities	3,76	0,91	0,74	0,90
CL13-The institution makes efforts to identify corporate vulnerabilities	3,88	0,89	0,78	0,90
CL14-The institution considers and plans for obvious vulnerabilities	3,92	0,86	0,75	0,90
CL15-The institution considers and plans for less obvious vulnerabilities	3,52	0,92	0,66	0,90
CL16-There are policies and procedures in the institution that consider the occurrence of undesirable situations	3,58	0,91	0,55	0,90
CL17-The institution’s leadership can make wise and rapid decisions	4,05	0,83	0,75	0,90
CL18-The institution’s leadership tends to abdicate its decision-making power during a crisis	2,67	1,43	0,16	0,92
CL19-The executive director asks for advice and opinion from the board and experts	3,85	1,00	0,56	0,90
CL20- During a crisis, the institution tends to deny the accusations or to say the least	2,18	1,20	-0,08	0,92
CL21-Leaders take actions that require courage	4,04	0,82	0,63	0,90
CL22-Leaders, in times of crisis, have high risk aversion	2,48	1,20	0,01	0,92
CL23-Leaders see the crisis as an opportunity and not a problem	3,39	1,10	0,30	0,91

Source: Research data

The corrected item-total correlations were all positive, except for item CL20 which was negatively correlated, therefore, there was clearly one deviant item. This item was deleted, and the Cronbach’s alpha coefficient calculated. The corrected item-total correlation coefficients were between 0.524 and 0.801, except for items CL18, CL22, and CL23 (0.106, -0.061, and 0.286, respectively). The alpha coefficients increase when these three items are deleted. These items were deleted, and 19 were left. Internal consistency was analyzed, calculating the Cronbach’s alpha coefficient for the remaining 19 items which indicated an overall coefficient r = 0.953, and Guttman split half coefficient = 0.881 (alpha part 1 = 0.925, alpha part 2 = 0.924).

Exploratory factor analysis (EFA) using the principal component method with varimax rotation was then carried out with the remaining items. The sample size is important in the factor analysis. Hair et al. (2010) suggest that the sample size would be at least 100. Given the sample size of 168, factors loading of 0.45 and higher will be considered significant for the interpretations proposed (Hair et al., 2010). In this round of EFA (Kaiser–Meyer–Olkin [KMO] = 0.929; Bartlett’s test of sphericity = 2585.8; df = 171; p-value < 0.001), two items (CL11 and CL17) with 0.5 or higher loadings on multiple factors were further removed (Chen et al., 2014). Afterwards, another round of EFA using the principal component method with varimax rotation (KMO = 0.915; Bartlett’s test of sphericity = 2191.35; df = 136; p-value < 0,001) was conducted with the 17 remaining items and three factors emerged and accounted for 69% of the variance, greater than the 60% reported by Hinkin (1998) as the minimum acceptable target (Table 5).

**Table 5:** Rotated factors loadings in the NPOs crisis leadership competencies

	Factors		
Items	1	2	3
CL1-The leadership team always communicates openly and honestly.	0,79		
CL2-The internal and external communication is explicit.	0,82		
CL3-Internal and external communication is sufficient.	0,71		
CL4-There is sharing of relevant information	0,76		
CL5-Employees feel safe in the work environment	0,68		
CL21-Leaders take actions that require courage	0,57		
CL12-The institution has identified all its vulnerabilities		0,71	
CL13-The institution makes efforts to identify corporate vulnerabilities		0,73	
CL14-The institution considers and plans for obvious vulnerabilities		0,79	
CL15-The institution considers and plans for less obvious vulnerabilities		0,81	
CL16-There are policies and procedures in the institution that consider the occurrence of undesirable situations		0,72	
CL19-The executive director asks for advice and opinion from the board and experts		0,54	
CL6-Clients or beneficiaries trust in the institution’s service			0,61
CL7-The institution’s partners expect cooperative actions and intention			0,61
CL8-The institution meets the needs of all stakeholders			0,82
CL9-The institution responds to all stakeholders			0,81
CL10-The institution is transparent and accountable to all stakeholders			0,75
**Eigenvalue**	9,23	1,43	1,07
**% of variance**	54,31	8,38	6,26
**Cronbach’s**	0,915	0,891	0,870

Source: Research data

The factorial model obtained after an EFA explains the structure of latent factors responsible for the correlations observed between the original variables (Maroco, 2018). The evaluation of the goodness of fit can be done through the Goodness of Fit Index (GFI), adjusted GFI and Root Mean Square Residual (RMSR). In our model GFI = 0.972152, which reveals a very good fit, adjusted GFI = 0.95 and RMSR = 0.055, that is, the model fit is good.

## Discussion

In the current study, based on James and Wooten’s (2005) seminal work, three dimensions are advanced as novelty, with several important implications for theory, practice, and future research.

### Theoretical Contributions

This study makes important theoretical contributions to the crisis leadership competencies literature on the NPOs context. To further develop this contribution, interpretation is needed which implies examining which variables are assigned to each factor and name it.

**Factor 1** entailed six items all with loadings of at least 0.57. The items included, CL1 (The leadership team always communicates openly and honestly), CL2 (The internal and external communication is explicit), CL3 (Internal and external communication is sufficient), CL4 (There is sharing of relevant information), CL5 (Employees feel safe in the work environment), and CL21 (Leaders take actions that require courage). The factor could be termed as “**Building a foundation of trust through communication**”. These results confirm arguments from James and Wooten’s (2005) two competencies (building a foundation of trust, and taking courageous action), and so, to build trust leaders need to communicate openly, honestly, and often, and must manage expectations through explicit communication, approaching crisis as an opportunity. These results link several arguments that highlight the importance of an open, two-way, communication to build relationships (Urick et al., 2021), nurture trust through a constant and open communication (Caringal-Go et al., 2021; Cohen et al., 2017; Jordan et al., 2016; Ulmer, 2012). Building trust and maintaining trust with the public is essential for NPOs and their reputation (Sisco, 2012). Crisis communication involves partnering with and understanding the public, collaborating with trusted sources, demonstrating accessibility to the media, sharing information honestly and openly, accepting uncertainty, and communicating self-efficacy messages (Seeger, 2006; Veil & Husted, 2012).

**Factor 2** entailed six items all with loadings of at least 0.54. The items included CL12 (The institution has identified all its vulnerabilities), CL13 (The institution makes efforts to identify corporate vulnerabilities), CL14 (The institution considers and plans for obvious vulnerabilities, CL15 (The institution considers and plans for less obvious vulnerabilities), CL16 (There are policies and procedures in the institution that consider the occurrence of undesirable situations), and CL19 (The executive director asks for advice and opinion from the board and experts). The factor can be called “**Planning based on identification of vulnerabilities”**. These results confirm arguments from James and Wooten’s (2005) (identifying the (not so) obvious firm vulnerabilities). Other authors add that decisiveness of the leader demonstrated through a solution-oriented approach appeared to help them immediately respond to the crisis (Caringal-Go et al., 2021). In this scenario, leaders that dominate group organization, problem-solving, strategic guidance, and decision-making with an ethical orientation are more prepared to navigate threats and unfamiliar circumstances (Fener & Cevik, 2015; Urick et al., 2021). A leader can never anticipate all crisis scenarios but should consider and plan for many of the obvious and a few of the less obvious threats (James & Wooten, 2005).

**Factor 3** entailed four items with loadings of at least 0.61. The items include CL6 (Clients or beneficiaries trust the institution’s service), CL7 (The institution’s partners expect cooperative actions and intention), CL8 (The institution meets the needs of all stakeholders), CL9 (The institution responds to all stakeholders), and CL10 (The institution is transparent and accountable to all stakeholders). The factor could be termed as “**Responding to all stakeholders through accountability**”. These results confirm arguments from James and Wooten’s (2005) one competency (creating a new corporate mindset). Indeed, NPOs are facing increased pressure for improved organizational practices that facilitate accountability to all stakeholders. During a crisis, the pressure for accountability increases as the organization, its stakeholders, and the community at large try to move from the crisis (Jordan et al., 2016), highlighting the importance of transparent communication between leaders and stakeholders (Cohen et al., 2017). This result corroborates that organizational leader are influenced by a number of external factors, and should take a big picture approach, meaning that they should see their organizations more completely, and recognize their responsibility and accountability to all stakeholders, considering multiple perspectives and the needs of various groups (James & Wooten, 2005).

Results show that promoting a new mindset that responds to all stakeholders through accountability (Factor 3) is very important (items means ranging from 3.96 to 4.44) with the factor mean value of 4.16. Equally, building a foundation of trust through an open and honest communication with several groups and entities (Factor 1) is quite relevant (items means ranging from 3.92 to 4.34) with the factor mean value of 4.155. Finally, Factor 2, highlights the importance of identifying vulnerabilities, obvious or not so obvious ones, and planning policies and procedures based on the experience of the board and expert opinions consultancy (items means ranging from 3.52 to 3.88) with the factor mean value of 3.751.

Additionally, in order to compare the responses by the two groups (technical directors versus executive directors), the factor scores were computed using the Regression Method, resulting in three standardized variables. The Mann-Whitney test was used, having verified the existence of statistically significant differences in Factor 3 (p-value = 0.01).

**Fig. 2:**
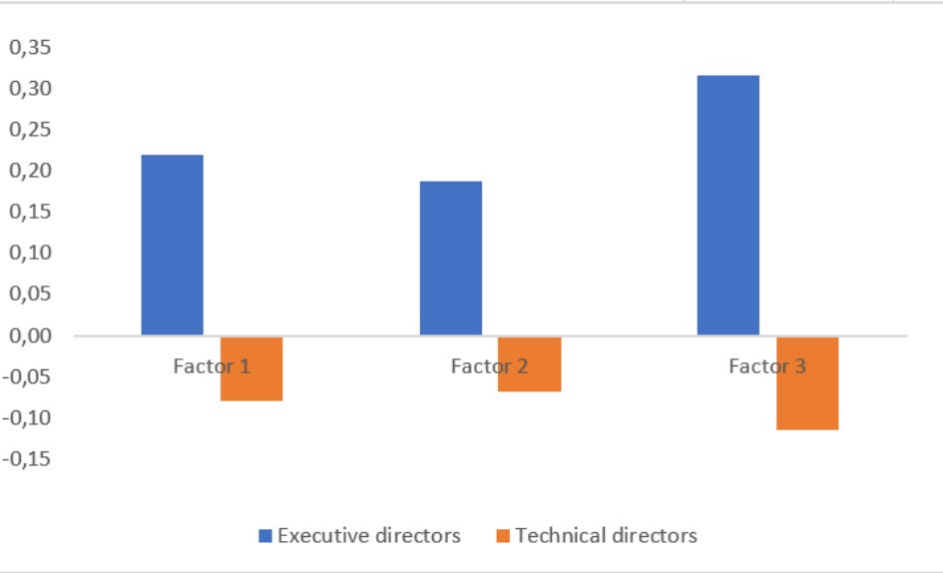
Comparison of the mean factors between the two groups. (Source: Research data)

This confirms the argument of Jaques (2012) and Mitroff and Pauchant (1990) stating the existence of a strong perception disagreement between top-management and technical functions in a company. It is possible to note that the group of executive directors assumes a higher average in all factors. Figure 2 presents the mean of each group for the three factors.

In sum, this research aims to contribute to the body of knowledge on organizational crisis leadership and crisis competency models. The leadership demonstrated throughout the process of managing a crisis differentiates NPOs. As open systems, NPOs must consider external and internal stakeholders by adding three streams of action: anticipate and plan through perceived weaknesses; build internal and external trust through open, honest, and ongoing communication; and finally, practice accountability to all stakeholders. A competency-based approach in NPOs will help to develop frameworks and models that point out the underlying characteristics of individuals as well as organizational standards leading to superior performance.

### Practical Implications

Crisis leadership differs from everyday leadership practices because it requires leaders to deal with the immediacy and complexity of the precipitating event and the uncertainty of the constantly changing circumstances as they lead their organization from response to recovery and beyond (Mutch, 2020: 70). The results of this study suggest that crisis in NPOs demand extra care, on the one hand, responding to all stakeholders with a transparent and accountable attitude. On the other hand, having a leadership that implements open and honest communication, builds trust, shares relevant information, promotes a safe internal environment, and takes courageous actions, which are all determinant to NPOs overcoming times of crises. Effective leadership during a crisis protects the organization’s stakeholders, builds trust, and assists in image maintenance (Jordan et al., 2016).

NPOs may respond through a variety of processes, like organizational control mechanisms, the introduction of performance management and measurement systems, and staff professionalization. The leaders need to develop several techniques in creating action plans, the necessary qualifications for the best possible crisis management and learn the ability to catch the signals of crisis and the consequent preparation and protection against the crisis. Leaders also need to maintain open, two-way, transparent, and ethical communication; focus on the collaborative process of defining shared values within their internal community; and, should attend to the well-being of personnel and the surrounding community. Developing strategy formulation tools and approaches will help NPOs leaders focus and rationally determine organizational goals and priorities, prepare and protect against crisis, develop action plans to address the concerns of stakeholders, refine existing mission statements, or develop long-term organizational plans.

## Conclusion

In closing, after a literature review, this study developed a survey to deepen our knowledge about organizational leadership competencies applied to NPOs in crises. Inspired by the six core competencies of James and Wooten (2005), three factors representing content similarities were extracted from 17 items using an exploratory factor analysis. Analysis yielded an excellent internal consistency and the model fit is good. In what concerns theoretical and management implications, most respondents consider that it is particularly important to respond to all stakeholders through accountability, plan based on identification of vulnerabilities, and build a foundation of trust through communication. This study also explores and confirms different perceptions of leadership competencies between intermediate employees and top management.

This research, aside from its exploratory nature, has some limitations, such as the questionnaires not having a balanced distribution by district in Portugal. The convenience sample is not representative of the population. A larger and broader sample would have deeper knowledge about this theme. Despite the limited nature of these results, most of the arguments of recent literature are confirmed and, specifically, James and Wooten (2005) proposal is partially confirmed, as this study was applied during the times of pre-crisis and crisis.

In the future, it will be interesting to explore how different communication strategies, actions, and performances during the COVID-19 pandemic impacted the image and reputation of NPOs. Comparative NPOs studies about different leadership competencies worldwide could also raise some cultural issues to be solved. Finally, it would be interesting to confirm these results in other international contexts and types of organizations. Additionally, in future research the concept of NPOs` organizational crisis leadership competencies during crisis should be consolidated by identifying and comparing the potential attributes and dimensions following the excellent advice of Podsakoff et al. (2016) and Houghton et al. (2022).

The conclusions of this study provide future researchers with a more accurate concept definition of organizational leadership competencies for crisis in NPOs and, possibly, enable them to develop and validate a measurement scale. This will be a critical tool for a deeper understanding of organizational issues and crisis management behaviors. Competency models within the context of nonprofit will be improved and developed based on this contribution.

## Data Availability

The identity of individuals from whom the data were obtained was kept strictly confidential. All respondents agreed to complete the study but did not agree for their data to be shared publicly, so supporting data is not available.

## Footnotes

1 The technical director is, in general, responsible for directing the facility, assuming responsibility for programming activities, and for coordinating, and supervising all staff, considering the need to establish the technical management model appropriate for the proper functioning of the facility.
